# Supplementary material for: Highly Sensitive and Durable Structured Fibre Sensors for Low-Pressure Measurement in Smart Skin
Source: Sensors (Basel). 2019 Apr 16;19(8):1811. doi: 10.3390/s19081811 (PMC6515294; doi:10.3390/s19081811)
Supplement: Supplementary file 1 [file sensors-19-01811-s001.pdf]

Highly Sensitive and Durable Structured Fibre Sensors for Low-Pressure Measurement in Smart Skin

Bao Yang [1], Su Liu [1], Xi Wang [2], Rong Yin [1], Ying Xiong [1] and Xiaoming Tao [1,*]

[1] Research Centre of Smart Wearable Technology, Nanotechnology Center of Functional and Intelligent Textiles and Apparel, Institute of Textiles and Clothing, The Hong Kong Polytechnic University, Hong Kong, China; bao.yang@polyu.edu.hk (B.Y.); suliu.liu@connect.polyu.hk (S.L.); rryin@polyu.edu.hk (R.Y.); ying-xy.xiong@connect.polyu.hk (Y.X.)

[2] Engineering Research Center of Digitized Textile & Apparel Technology, Ministry of Education, College of Information Science and Technology, Donghua University, Shanghai, China, 201620; xiwang@dhu.edu.cn

* Correspondence: xiao-ming.tao@polyu.edu.hk; Tel.: +852-2766-6470

S1. Theoretical treatment of structured fibre pressure sensor

The designed sensor shown in **Figure 1a** is comprising by matrix, optical fibre with fibre Bragg gratings, a spacer, and a rigid base with a rectangular groove. First, only mall deformation of all components occurs due to the fracture strain of silica is only 0.6% [1]. Accordingly, it is reasonable to assume that all components are made by the linear-elastic material. Secondly, the optical fibre is well fixed on the rigid base by using glues (Aron alpha). The stiffness of the base made of ABS or Invar are three orders higher than that of optical fibre or the silicone film. Coupled with the above announcements, the fixed optical fibre can be considered as an elastic beam with the built-in condition. Thirdly, the film over the groove is simplified as a simply-supported plate. Because only part of bottom surface of the film is fixed on the base, while the top surface of the film is free. Moreover, the thin film is normally soft and has very low elastic modulus comparing to that of the base. For those reasons, the thin film can be simplified as a simply-supported plate. Fourthly, the groove has a sufficient depth, so that the optical fibre will not touch the bottom of the groove during deformation. Fifthly, the influences of the spacer on the flexural stiffness on the optical fibre and the soft matrix film are neglected. As well as the deformation of spacer in the compression direction is also neglected. Moreover, the spacer has a sufficient thickness, so that the optical fibre does not contact with the thin film on the zone over

the groove. Sixthly, the wavelength of FBGs induced by the pressure applied from the spacer can be neglected comparing to that inducing by axial deformation. To construct the theoretical model, several assumptions are made. From these assumptions, three cases will be included: a simply-supported rectangular plate with a load uniformly distributed over a rectangle zone shown in **Figure 1b**, which is corresponding to the shape of the spacer, a simply-supported rectangular plate under a uniform pressure shown in **Figure 1c**, and a built-in beam under a uniform load over the center part shown in **Figure 1d**, corresponding to the length of the spacer.

S1.1 Theoretical Treatments of a Simply-Supported Plate under Uniform Pressure

Let us firstly consider the case of a simply-supported rectangular plate under a uniform load, p_1, distributed over a shaded rectangle (corresponding to the spacer) with the sides of a_s and b_s, shown in **Figure 1b**. The derivation of deflection, W_1, is similar to that shown in reference [2], having a different mathematic form because the origins of coordinates are different. The deflection, W_1, at any point of the plate is given below.

$$W_1 = \frac{4p_1a^4}{D\pi^5}\sum_{m=1,3,5,\cdots}^{\infty}\frac{1}{m^5}\sin\frac{m\pi a_s}{2a}\left\{1-\frac{\cosh\frac{m\pi y}{a}}{\cosh\alpha_m}\left[\cosh\left(\alpha_m-2\gamma_m\right)+\gamma_m\sinh\left(\alpha_m-2\gamma_m\right)+\alpha_m\frac{\sinh 2\gamma_m}{2\cosh\alpha_m}\right]+\frac{\cosh\left(\alpha_m-2\gamma_m\right)}{2\cosh\alpha_m}\frac{m\pi y}{a}\sinh\frac{m\pi y}{a}\right\}\cos\frac{m\pi x}{a} \tag{S1-1}$$

where $\alpha_m = \frac{m\pi b}{2a}$, $\gamma_m = \frac{m\pi b_s}{4a}$, $D = \frac{Eh^3}{12(1-\mu^2)}$, E, μ, and h are the elastic modulus, poison's ratio and thickness of the plate, a and b are the length of the rectangular plate in x direction and the width of the rectangular plate in y direction, respectively, corresponding to the size of the groove on the base.

Similarly, as shown in **Figure 1c**, a uniform load, *p*, is distributed over the whole rectangle, meaning $a_s = a$ and $b_s = b$, thus, the deflection, W_2, at any point of the plate can be given by

$$W_2 = \frac{4pa^4}{D\pi^5} \sum_{m=1,3,5,\cdots}^{\infty} \frac{(-1)^{(m-1)/2}}{m^5} \left(1 - \frac{\alpha_m \tanh\alpha_m + 2}{2\cosh\alpha_m} \cosh\frac{2\alpha_m y}{b} + \frac{\alpha_m}{\cosh\alpha_m} \frac{y}{b} \sinh\frac{2\alpha_m y}{b}\right) \cos\frac{m\pi x}{a} \qquad \text{(S1-2)}$$

S1.2. Theoretical Treatments of a Built-in Beam under a Load Uniformly Distributed at the Center Part

As shown in **Figure 1d**, a load, *q*, is uniformly distributed at the center part of a built-in beam. The origin of a coordinate *x* is set at the center of the beam. The deflection can be obtained by sequential integration of the basic relations. First, the applied load, *F*, from the spacer, is given by

$$F = \begin{cases} 0, & -\frac{a}{2} \le x < -\frac{a_s}{2} \\ -q, & -\frac{a_s}{2} \le x \le \frac{a_s}{2} \\ 0, & \frac{a_s}{2} < x \le \frac{a}{2} \end{cases} \qquad \text{(S1-3)}$$

Next, the shear force, *Q*, is obtained by integration of the transverse equilibrium equation, $\frac{dQ}{dx} = F$.

$$Q = \begin{cases} \frac{qa_s}{2}, & -\frac{a}{2} \le x < -\frac{a_s}{2} \\ -qx, & -\frac{a_s}{2} \le x \le \frac{a_s}{2} \\ -\frac{qa_s}{2}, & \frac{a_s}{2} < x \le \frac{a}{2} \end{cases} \qquad \text{(S1-4)}$$

Next, from $\frac{dM}{dx} = Q$, the moment, *M*, neglecting the influence of force in *x*-axial direction on the moment due to small deformation, is given by

$$M = \begin{cases} \dfrac{qa_s x}{2} + C_0 + \dfrac{qa_s^{\,2}}{8}, & -\dfrac{a}{2} \le x < -\dfrac{a_s}{2} \\ -\dfrac{qx^2}{2} + C_0, & -\dfrac{a_s}{2} \le x \le \dfrac{a_s}{2} \\ -\dfrac{qa_s x}{2} + C_0 + \dfrac{qa_s^{\,2}}{8}, & \dfrac{a_s}{2} < x \le \dfrac{a}{2} \end{cases} \tag{S1-5}$$

where the arbitrary constant C_0 is the moment at the center of the beam.

Then, from $\dfrac{d\theta}{dx} = \dfrac{M}{E_b I_z}$, the rotation, θ, gives

$$\theta = \frac{1}{E_b I_z} \begin{cases} \dfrac{qa_s x^2}{4} + \left(C_0 + \dfrac{qa_s^{\,2}}{8} \right) x + C_1 + \dfrac{qa_s^{\,3}}{48}, & -\dfrac{a}{2} \le x < -\dfrac{a_s}{2} \\ -\dfrac{qx^3}{6} + C_0 x + C_1, & -\dfrac{a_s}{2} \le x \le \dfrac{a_s}{2} \\ -\dfrac{qa_s x^2}{4} + \left(C_0 + \dfrac{qa_s^{\,2}}{8} \right) x + C_1 - \dfrac{qa_s^{\,3}}{48}, & \dfrac{a_s}{2} < x \le \dfrac{a}{2} \end{cases} \tag{S1-6}$$

where E_b and I_z are the elastic modulus and the cross-sectional moment of inertia of the beam, respectively.

From the symmetry condition of $\theta(x=0)=0$, an arbitrary constant C_1 can be eliminated, that is, $C_1 = 0$. Then, the moment, C_0, is determined by another boundary condition, $\theta(x=-a/2)=0$, whereby $C_0 = \dfrac{qa_s^{\,3}}{24a} + \dfrac{qa_s a}{8} - \dfrac{qa_s^{\,2}}{8}$. Merging C_0 and C_1 into equation (S1-5) and (S1-6), M and [illegible] can be presented as

$$M = \begin{cases} \dfrac{qa_s x}{2} + \dfrac{qa_s a}{8} + \dfrac{qa_s^{\,3}}{24a}, & -\dfrac{a}{2} \le x < -\dfrac{a_s}{2} \\ -\dfrac{qx^2}{2} + \dfrac{qa_s a}{8} + \dfrac{qa_s^{\,3}}{24a} - \dfrac{qa_s^{\,2}}{8}, & -\dfrac{a_s}{2} \le x \le \dfrac{a_s}{2} \\ -\dfrac{qa_s x}{2} + \dfrac{qa_s a}{8} + \dfrac{qa_s^{\,3}}{24a}, & \dfrac{a_s}{2} < x \le \dfrac{a}{2} \end{cases} \tag{S1-7}$$

$$\theta = \frac{1}{E_b I_z}\begin{cases} \frac{qa_s x^2}{4} + \left(\frac{qa_s a}{8} + \frac{qa_s^3}{24a}\right)x + \frac{qa_s^3}{48}, & -\frac{a}{2} \le x < -\frac{a_s}{2} \\ -\frac{qx^3}{6} + \left(\frac{qa_s a}{8} + \frac{qa_s^3}{24a} - \frac{qa_s^2}{8}\right)x, & -\frac{a_s}{2} \le x \le \frac{a_s}{2} \\ -\frac{qa_s x^2}{4} + \left(\frac{qa_s a}{8} + \frac{qa_s^3}{24a}\right)x - \frac{qa_s^3}{48}, & \frac{a_s}{2} < x \le \frac{a}{2} \end{cases} \quad \text{(S1-8)}$$

Finally, the deflection can be given by the integration of the shear and moment.

$$\frac{dW}{dx} = -\theta + \gamma \quad \text{(S1-9)}$$

where $\gamma = \frac{Q}{GA_z}$, G and A_z are the shear rigidity and the cross-sectional area of the beam, respectively. From which, the deflection, W_3, at any point of the beam is given by

$$W_3 = \begin{cases} \frac{-1}{E_b I_z}\left[\frac{qa_s x^3}{12} + \left(\frac{qa_s a}{8} + \frac{qa_s^3}{24a}\right)\frac{x^2}{2} + \frac{qa_s^3 x}{48} + C_2 + \frac{qa_s^4}{384} - \frac{qa_s^2 E_b I_z}{8GA_z}\right] + \frac{qa_s x}{2GA_z}, & -\frac{a}{2} \le x < -\frac{a_s}{2} \\ \frac{-1}{E_b I_z}\left[-\frac{qx^4}{24} + \left(\frac{qa_s a}{8} + \frac{qa_s^3}{24a} - \frac{qa_s^2}{8}\right)\frac{x^2}{2} + C_2\right] - \frac{qx^2}{2GA_z}, & -\frac{a_s}{2} \le x \le \frac{a_s}{2} \\ \frac{-1}{E_b I_z}\left[-\frac{qa_s x^3}{12} + \left(\frac{qa_s a}{8} + \frac{qa_s^3}{24a}\right)\frac{x^2}{2} - \frac{qa_s^3 x}{48} + C_2 + \frac{qa_s^4}{384} - \frac{qa_s^2 E_b I_z}{8GA_z}\right] - \frac{qa_s x}{2GA_z}, & \frac{a_s}{2} < x \le \frac{a}{2} \end{cases}$$

(S1-10)

The arbitrary constant, C_2, is determined by the boundary, $W_3(x = -a/2) = 0$, whereby

$$C_2 = -\frac{E_b I_z qa_s a}{4GA_z} - \frac{qa_s a^3}{192} + \frac{qa_s^2 E_b I_z}{8GA_z} + \frac{qa_s^3 a}{192} - \frac{qa_s^4}{384}.$$

S1.3. Evaluation of Average Axial Strain of the Built-in Beam

After fabrication, the plate and the beam have an initial deflection due to a spacer is setup between the plate and the beam. At the initial condition, $p_{s,I}$ and $q_{s,I}$, represent the effective interactive force between the spacer and the plate and the fibre, respectively. Based on compatibility condition of displacement and force, one gives

$$\begin{cases} -p_{s,\mathrm{I}} W_{1,e} + q_{s,I} W_{3,e} = h_s \\ p_{s,\mathrm{I}} b_s = q_{s,I} \end{cases} \quad \text{(S1-11)}$$

where h_s is the thickness of the spacer. $W_{1,e}$ and $W_{3,e}$ are the effective deflection of the plate under unit load uniformly distributed over the rectangular area of the spacer and the effective deflection of the beam under unit load distributed over the center part covered by the spacer, respectively.

$$W_{1,e} = \frac{\int_{-b_s/2}^{b_s/2} \int_{-a_s/2}^{a_s/2} W_1 \left(p_1 = 1 \right) dxdy}{a_s b_s} \quad \text{(S1-12)}$$

$$W_{3,e} = \frac{\int_{-a_s/2}^{a_s/2} W_3 \left(q = 1 \right) dx}{a_s} \quad \text{(S1-13)}$$

Then, $p_{s,I}$ and $q_{s,I}$ can be given by

$$p_{s,\mathrm{I}} = \frac{h_s}{W_{1,e} + b_s \times W_{3,e}} \quad \text{(S1-14)}$$

$$q_{s,I} = \frac{h_s \times b_s}{W_{1,e} + b_s \times W_{3,e}} \quad \text{(S1-15)}$$

If an external load, *p*, applied on the top surface of the plate, the compatibility conditions of displacement and force are also satisfied, and the effective contact force from the spacer on the plate and the fibre becomes p_s and q_s, respectively.

$$\begin{cases} p \times W_{2,e} - W_{1,e} \times \left(p_s - p_{s,I} \right) = W_{3,e} \times \left(q_s - q_{s,I} \right) \\ p_s b_s = q_s \end{cases} \quad \text{(S1-16)}$$

where $W_{2,e}$ is given by

$$W_{2,e} = \frac{\int_{-b_s/2}^{b_s/2} \int_{-a_s/2}^{a_s/2} W_2 \left(p = 1 \right) dxdy}{a_s b_s} \quad \text{(S1-17)}$$

From the above equation system, p_s and q_s can be solved.

$$p_s = \frac{p \times W_{2,e} + h_s}{W_{1,e} + b_s \times W_{3,e}} \quad \text{(S1-18)}$$

$$q_s = \frac{\left(p \times W_{2,e} + h_s \right) \times b_s}{W_{1,e} + b_s \times W_{3,e}} \quad \text{(S1-19)}$$

Then from equation (S10), the deflection, W, of the optical fibre induced by an external load can be given by

$$W=\begin{cases}\dfrac{-1}{E_bI_z}\left[\dfrac{q_sa_sx^3}{12}+\left(\dfrac{q_sa_sa}{8}+\dfrac{q_sa_s{}^3}{24a}\right)\dfrac{x^2}{2}+\dfrac{q_sa_s{}^3x}{48}+C_2+\dfrac{q_sa_s{}^4}{384}-\dfrac{q_sa_s{}^2E_bI_z}{8GA_z}\right]+\dfrac{q_sa_sx}{2GA_z}, & -\dfrac{a}{2}\le x<-\dfrac{a_s}{2}\\ \dfrac{-1}{E_bI_z}\left[-\dfrac{q_sx^4}{24}+\left(\dfrac{q_sa_sa}{8}+\dfrac{q_sa_s{}^3}{24a}-\dfrac{q_sa_s{}^2}{8}\right)\dfrac{x^2}{2}+C_2\right]-\dfrac{q_sx^2}{2GA_z}, & -\dfrac{a_s}{2}\le x\le\dfrac{a_s}{2}\\ \dfrac{-1}{E_bI_z}\left[-\dfrac{q_sa_sx^3}{12}+\left(\dfrac{q_sa_sa}{8}+\dfrac{q_sa_s{}^3}{24a}\right)\dfrac{x^2}{2}-\dfrac{q_sa_s{}^3x}{48}+C_2+\dfrac{q_sa_s{}^4}{384}-\dfrac{q_sa_s{}^2E_bI_z}{8GA_z}\right]-\dfrac{q_sa_sx}{2GA_z}, & \dfrac{a_s}{2}<x\le\dfrac{a}{2}\end{cases}$$

(S1-20)

Finally, the average strain, , along axial direction can be estimated by integration of the deflection.

$$\varepsilon=\left(\int_{-a/2}^{a/2}W dx-a\right)/a \qquad \text{(S1-21)}$$

S1.4. Evaluation of the Wavelength Shift of FBGs

The typical optical fibre with fibre Bragg gratings is shown in **Figure 1a**. The fibre has multiple layers, including core, cladding and coating layers. The mechanical properties of the layers normally are different, such as the elastic modulus of the core layer and the cladding layer (~ 70 GPa) is higher that of the coating layer (~ 2.5 GPa) for the optical fibre utilized in this study. The effective parameters of the beam can be estimated by the combination among the elastic modulus, the cross-sectional moment inertia and the cross-sectional area, which are given below.

$$E_bI_z=E_{c1}\frac{\pi d_{c1}{}^4}{64}+E_{c2}\frac{\pi\left(d_{c2}{}^4-d_{c1}{}^4\right)}{64}+E_{c3}\frac{\pi\left(d_{c3}{}^4-d_{c2}{}^4\right)}{64} \qquad \text{(S1-22)}$$

$$E_bA_z=E_{c1}\frac{\pi d_{c1}{}^2}{4}+E_{c2}\frac{\pi\left(d_{c2}{}^2-d_{c1}{}^2\right)}{4}+E_{c3}\frac{\pi\left(d_{c3}{}^2-d_{c2}{}^2\right)}{4} \qquad \text{(S1-23)}$$

$$GA_z=\frac{E_{c1}}{2\left(1+\mu_{c1}\right)}\frac{\pi d_{c1}{}^2}{4}+\frac{E_{c2}}{2\left(1+\mu_{c2}\right)}\frac{\pi\left(d_{c2}{}^2-d_{c1}{}^2\right)}{4}+\frac{E_{c3}}{2\left(1+\mu_{c3}\right)}\frac{\pi\left(d_{c3}{}^2-d_{c2}{}^2\right)}{4} \qquad \text{(S1-24)}$$

where E_{c1}, μ_{c1}, and d_{c1} are the elastic modulus, Poisson's ratio and diameter of the core layer, respectively. E_{c2} , μ_{c2} , and d_{c2} are the elastic modulus, Poisson's ratio and diameter of the cladding layer, respectively, E_{c3}, μ_{c3}, and d_{c3} are the elastic modulus, Poisson's ratio and diameter of the coating layer, respectively.

Then merging equations (S1-22), (S1-23) and (S1-24) into the above relations, replacing the $E_b I_z$, $E_b A_z$ and GA_z . And the average axial strain of FBGs can be obtained from equation (S1-21). The Bragg wavelength, λ_B ,of fibre Bragg gratings is sensitive to the axial strain and temperature. The wavelength shift, $\Delta\lambda$, induced by the applied strain, ε , and change of temperature, ΔT , can be approximately given by[3]

$$\Delta\lambda = \left(C_s \varepsilon + C_T \Delta T\right) \lambda_B \qquad \text{(S1-25)}$$

where λ_B is the initial Bragg wavelength of FBGs, C_s is the coefficient of the applied strain, and C_T is the coefficient of temperature, which is made up of the thermal expansion coefficient and the thermos-optic coefficient. If the temperature of the FBG pressure sensors is constant, the equation (S1-25) can be simplified by

$$\Delta\lambda = C_s \varepsilon \lambda_B \qquad \text{(S1-26)}$$

S2. Mechanical properties of Silicone 903

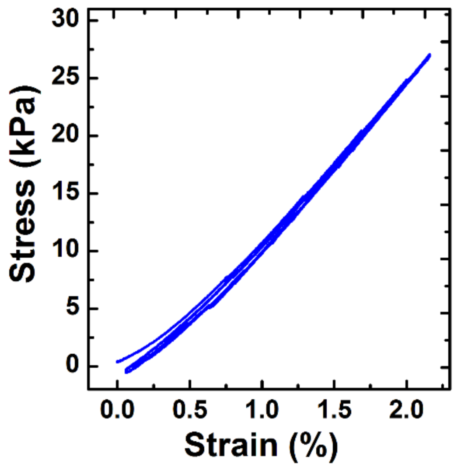

Figure S1. Stress-strain curves of a cubic specimen made of silicone 903, which is laboratory-fabricated in the length of sides of 2 cm. The test was carried out on the machine (Instron 5566, USA), and the loading rate applied on the specimen is set up of 20 N/min, including 3cycles.

As shown in **Figure S1**, stress-strain cures of an illustrated specimen made of silicone 903 indicates that such silicone has good elasticity, an approximately linear relationship between stress and strain with low hysteresis (~ 2.1% Full Scale Output). The effective elastic modulus in the studied range is about 1.3 MPa.

Reference

1. Ang, J.; Li, H.C.H.; Herszberg, I.; Bannister, M.K.; Mouritz, A.P. Tensile fatigue properties of fibre Bragg grating optical fibre sensors. *International Journal of Fatigue* **2010**, *32*, 762-768.
2. Timoshenko, S.P.; Woinowsky-Krieger, S. *Theory of plates and shells*; McGraw-hill: 1959.
3. Othonos, A.; Kalli, K. Fiber Bragg Gratings: Fundamentals and Applications in Telecommunications and Sensing (Artech House Optoelectronics Library). *Artech House, Boston, London* **1999**.
